# Supplementary material for: Usefulness of Contrast-Enhanced Ultrasound in the Differentiation between Hepatocellular Carcinoma and Benign Liver Lesions
Source: Diagnostics (Basel). 2023 Jun 10;13(12):2025. doi: 10.3390/diagnostics13122025 (PMC10297446; doi:10.3390/diagnostics13122025)
Supplement: Supplementary file 1 [file diagnostics-13-02025-s001.zip › diagnostics-2439437-supplementary.pdf]

Table S1. Demographic and size of lesion analysis. Mean ± SD values supplementary (excluded from manuscript).

**Patient characteristics**	**FNH**	**HCA**	**FNH+HCA**	**HCC**
Sex				
Male	0	6	6	21
Female	11	12	23	7
Age				
Range	22 - 41	19 - 80	19 - 80	39 - 85
Mean ± SD	32 ± 5.7	37.2 ± 16.7	35.2 ± 13.7	65.2 ± 11.7
Smaller dimension of a lesion (CEUS) [cm]				
Range	0.9-3.6	0.62 - 7.69	0.62 - 7.69	1.59 - 16.61
Mean ± SD	2.43 ± 0.83	2.71 ± 1.70	2.59 ± 1.42	5.76 ± 3.01
Smaller dimension of a lesion (CEUS) [cm]				
Range	1.41 - 4.6	0.66 - 8.2	0.66 - 8.2	2.88 - 18.63
Mean ± SD	3.20 ± 0.90	3.31 ± 1.83	3.27 ± 1.52	7.22 ± 3.34
